# A New Disaster Information Sensing Mode: Using Multi-Robot System with Air Dispersal Mode

**DOI:** 10.3390/s18103589

**Published:** 2018-10-22

**Authors:** Yi Liu, Junyao Gao, Jingchao Zhao, Xuanyang Shi

**Affiliations:** 1 Intelligent Robotics Institute, School of Mechatronical Engineering, Beijing Institute of Technology, Beijing 100081, China; YiLiu@bit.edu.cn (Y.L.); jch_zhao@yeah.net (J.Z.); 3120170111@bit.edu.cn (X.S.); 2 Key Laboratory of Biomimetic Robots and Systems, Ministry of Education, Beijing 100081, China; 3 Key Laboratory of Intelligent Control and Decision of Complex System, Beijing Institute of Technology, Beijing 100081, China; 4 Beijing Institute of Control Engineering, Beijing 100081, China

**Keywords:** mobile robot, remote sensing, disaster response, air deployment, natural hazards

## Abstract

This paper presents a novel sensing mode for using mobile robots to collect disaster ground information when the ground traffic from the rescue center to disaster site is disrupted. Traditional sensing modes which use aerial robots or ground robots independently either have limited ability to access disaster site or are only able to provide a bird’s eye view of the disaster site. To illustrate the proposed sensing mode, the authors have developed a Multi-robot System with Air Dispersal Mode (MSADM) by combining the unimpeded path of aerial robots with the detailed view of ground robots. In the MSADM, an airplane carries some minimal reconnaissance ground robots to overcome the paralyzed traffic problem and deploys them on the ground to collect detailed scene information using parachutes and separation device modules. In addition, the airplane cruises in the sky and relays the control and reported information between the ground robots and the human operator. This means that the proposed sensing mode is able to provide more reliable communication performance when there are obstacles between the human operators and the ground robots. Additionally, the proposed sensing mode can easily make use of different kinds of ground robots, as long as they have a compatible interface with the separation device. Finally, an experimental demonstration of the MSADM is presented to show the effectiveness of the proposed sensing mode.

## 1. Introduction

In disasters, victim mortality drastically increases after 48 h due to the lack of food and medical treatment. Therefore, it is vital to collect information in disaster zones for situation assessment and to develop a suitable rescue plan [1,2]. In particular, there is an increased need for disaster sensing modes to gather information on the disaster site when ground traffic is disrupted by the disaster, since it is difficult for human rescuers to access the disaster zone in such cases.

**Figure 1:**
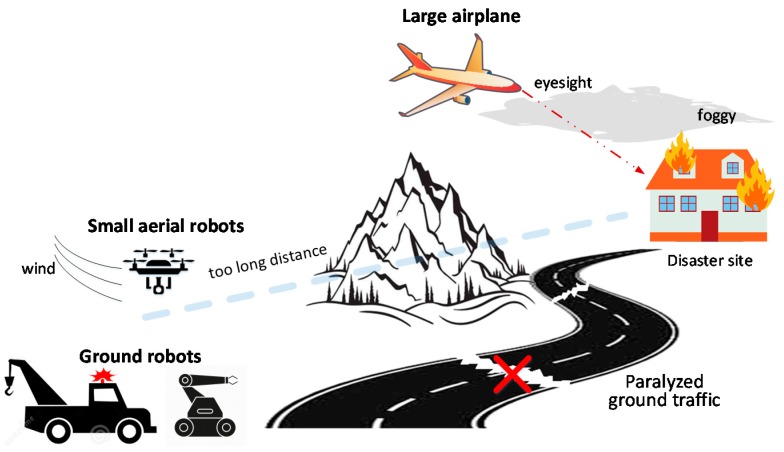
Limitations of traditional sensing modes under paralyzed ground traffic situations.

Historically, traditional sensing modes have used ground or aerial robots independently to enter the disaster area and relay disaster information back to human operators. This sensing mode has been adopted in many applications and has been certificated to be effective in some applications: ground robots are used to find trappers [3] and monitor environments [4]; unmanned aerial vehicles play important roles in wilderness search and rescue activities [5,6,7,8]. However, it is difficult to collect ground information in paralyzed ground traffic situations due to the inherent limitations of ground robots and aerial robots. Ground search and rescue robots can collect ground information in detail if they have been brought to the vicinity of the disaster scene. While under paralyzed road traffic conditions caused by the disaster, ground robots tend to have limited ability to access the disaster site, let alone collect disaster scene information (see Figure 1). Aerial robots are a good choice for disaster response when ground traffic is disrupted, owing to their advantages of high speed and the unimpeded air path. However, there are still some shortcomings in such situations. Large military airplanes such as the Ikhana Global Hawk cannot collect details like ground robots, and only provide a bird’s eye view of disaster scenes [9]. Moreover, the performance can easily suffer from poor visibility caused by foggy weather. Small aerial robots like quadrotors have to rotate their propellers all the time to counteract their own weight, leading to a short endurance time. Thus, they are unable to cover long distances or provide continuous information collection like ground robots. Moreover, they are easily affected by wind and need to be carefully controlled to avoid damage (see Figure 1).

**Figure 2:**
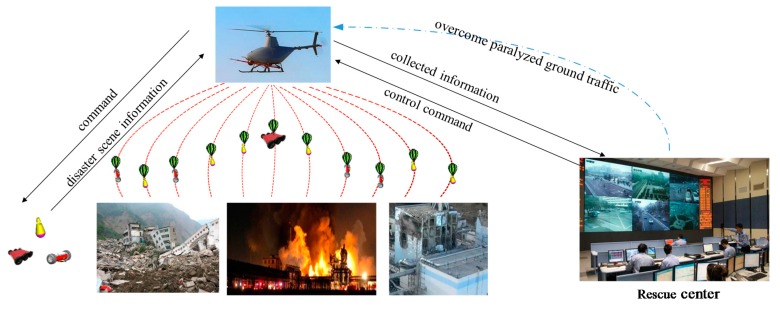
Disaster sensing mode using MSADM.

The main aim of this paper is to provide a new sensing mode for collecting ground information on disaster sites when ground traffic is disrupted. Motivated by the pros and cons of ground and aerial robots, a new sensing mode is proposed in this paper (See Figure 2). Instead of using ground or aerial robots independently in the traditional sensing mode, the proposed sensing mode combines the aerial robots’ unimpeded air path with the ground robots’ detailed view. To illustrate the proposed sensing mode, the authors have developed a Multi-robot System with Air Dispersal Mode (MSADM). In the MSADM, an airplane carries some minimal reconnaissance ground robots in order to overcome the paralyzed traffic problem and deploys them to the ground to collect detailed scene information using parachutes and separation device modules. Meanwhile, the airplane cruises in the sky and relays the control and reported information between the ground robots and the human operator.

The main advantage of the proposed sensing mode is that it can collect detailed ground information when road traffic is disrupted. In addition, the robots’ communication performance is enhanced by using the airplane as a communication relay between the ground robots and the human operator. Furthermore, the proposed sensing mode can easily make use of different kinds of ground robot, as long as they have an interface that is compatible with the separation device.

The rest of this paper is organized as follows. Section 2 describes related research. Section 3 introduces the proposed sensing mode for collecting ground information at the disaster site when road traffic is disrupted. In Section 4, the experimental platform for verification of the proposed sensing mode is presented. An integrated demonstration is conducted in Section 5. Section 6 is the summary and conclusion.

## 2. Related Research

Studies about disaster information sensing can be found in rescue projects and disaster robots. Ground robots with video transmission ability can replace human rescuers to gather disaster information without the need for rescuers to risk their lives. Deployable miniature reconnaissance robots like the “Recon Scout” and “FirstLook 110” were developed to be tossed into terrorism disaster scenes for terrorist surveillance [10,11]. Portable tracked robots such as the “iRobot Packbot” and “NIFI” are frequently used to search for survivors and for mapping building structures after disasters [12,13]. Robots equipped with manipulators have been used in mine disasters to clear obstacles and provide a unique camera viewpoint in the disaster [14,15]. Non-wheeled robots such as “Rhex” and “Terminator bot”, who use their legs to climb random steps, have shown great potential in disaster response [16,17]. However, these ground robots can gather disaster information only if they have been brought to the vicinity of the disaster scene. Meanwhile, under paralyzed road traffic circumstance caused by the disaster, ground robots tend to have limited ability to access the disaster site, let alone collect disaster scene information.

Aerial robots have drawn researchers’ attention due to their fast speed and ability to enter disaster areas directly. Military aircraft have been used to transport soldiers to disaster sites after earthquakes, and to monitor fire in the western states of the US [9]. Small UAVs with image-sensing abilities also play an important role in search and rescue missions [18,19]. However, they are unable to collect ground information as detailed as ground robots, since they observe the environment form the air. Moreover, small UAVs are also easily affected by the wind and are unsuitable for covering long distances or times due to their short endurance time.

In recent years, disaster-sensing modes which use ground robots and aerial robots together have been proposed. Hsieh, M.A. used a team of ground and aerial robots for situational awareness of a small village [20]. Three ground robots and an aerial robot were used cooperatively to build a map of a building damaged in the 2011 Tohoku earthquake Sendai (Japan) [21]. However, these studies assumed that the ground robots could be brought to the vicinity of the disaster site following the occurrence of a disaster. In fact, ground traffic is not guaranteed in the disaster events.

## 3. The Proposed Sensing Mode: Using MSADM for Disaster Sensing

The main idea of the proposed sensing mode is to integrate the advantages of aerial robots’ direct access to the disaster site and ground robots’ detailed view into a single robotic system (MSADM), and use it for disaster sensing. In the following subsections, the proposed sensing mode and MSADM will be carefully illustrated to solve the problem of gathering disaster information when ground traffic is disrupted. Firstly, the overall architecture of the proposed mode and its advantages are derived. Then the communication link of the MSADM is presented. Finally, the separation device which facilitates the versatility and scalability of the proposed sensing mode is introduced.

### 3.1. Architecture of the Proposed Sensing Mode

In 2008, during China’s Great Sichuan earthquake, the persistent heavy rain and landslides occurring after the earthquake blocked road traffic, and rescue teams were not able to access the disaster area for collection of disaster ground information. In this, 15 paratroopers risked their lives, jumping from 5000 m into the worst-hit area, Mao County, to provide awareness of the disaster situation [22]. Motivated by this event, the concept of using an airplane to carry miniature reconnaissance ground robots to overcome the paralyzed traffic problem and deploy them to the ground for detailed information collection is proposed.

**Figure 3:**
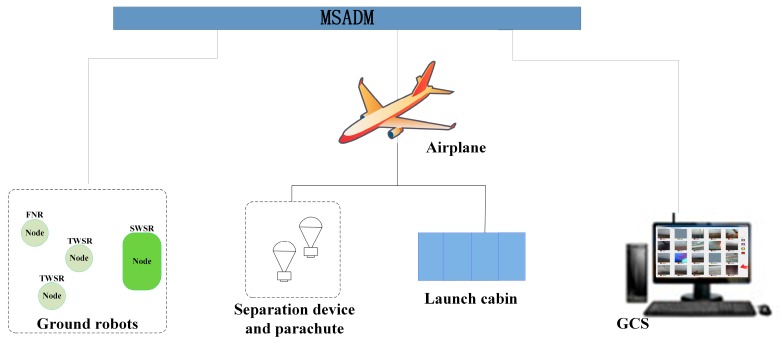
Schematic diagram of the MSADM.

In this paper, MSADM is designed by the authors to implement this concept. Figure 3 shows the schematic diagram of MSADM. Besides the airplane and ground robots, a ground control station (GCS) is provided for interaction between system and users. A launch cabin is equipped on the airplane to load and deploy the ground robots. A parachute and separation device is used here to help the deployment of ground robots. In detail, an object is easily broken into pieces when falling freely from high altitude without any protection. The separation device connects the robot with a parachute to slow the robot’s velocity during deployment and reduce the shock when landing. Furthermore, the separation device separates itself from the ground robots to enable the free movement of the ground robot after it recognizes that the robot has landed on the ground.

**Figure 4:**
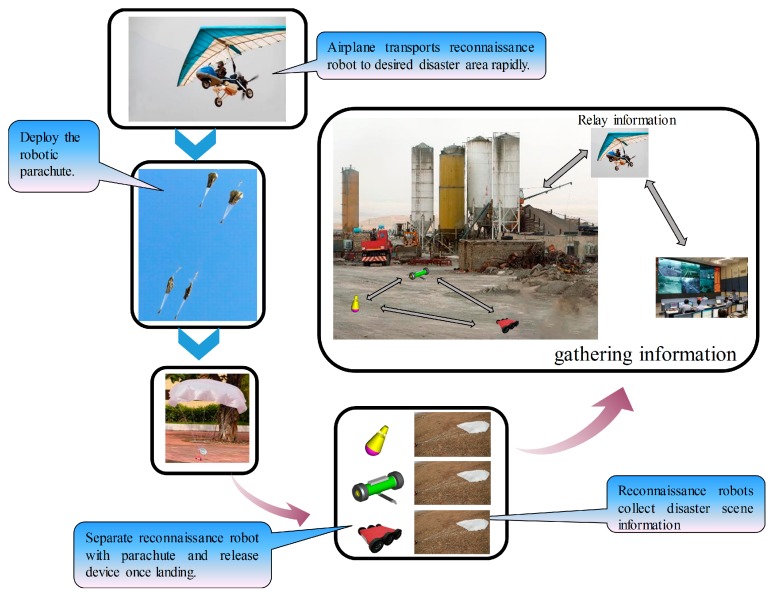
Flow chart of the proposed sensing mode.

Figure 4 shows the flow chart of the proposed sensing mode: using the MSADM for disaster sensing. Firstly, the airplane is used to overcome the paralyzed traffic problem and transport ground robots to the desired area as soon as possible. After the delta-wing aircraft arrives at the desired disaster site, ground robots will be deployed from the air. In detail, each ground robot is connected to a parachute, together with a separation device, to avoid crashing into the ground at high velocity. The automatic separation module detects the acceleration changes during the deployment. Once the separation device recognizes that the robot has landed, it separates the robot from the parachute to enable the robot’s free movement. Finally, each ground robot is used to collect detailed ground information at the disaster site and report it to the GCS through the relay of the airplane. In this way, the new proposed sensing mode is able to collect ground information on the disaster site even when the ground traffic is disrupted.

### 3.2. Communication Link

The communication link of disaster robotic systems is always a thing that needs to be carefully handled in disaster response applications. Traditional disaster robots communicate directly with human users, and the communication performance suffers from the obstacles between them. One possible way to solve this problem is to add communication relays between them when the direct communication cannot be established [23].

**Figure 5:**
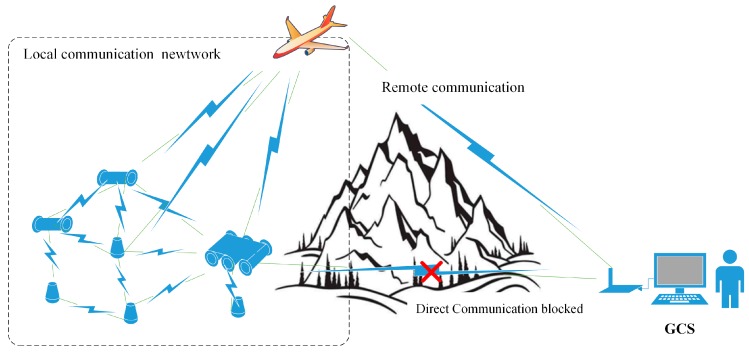
Communication link of the MSADM.

In the communication link of MSADM, the airplane cruises in the sky and acts as a relay between the ground robots and GCS. By doing this, the blocked ground communication link between the GCS and ground robots is transformed into parts of a barrier-free ground-air communication: namely, “remote communication” between the airplane and the GCS, and a “local communication network” among the ground robots and the airplane (see Figure 5).

Remote communication is defined as the communication between the ground robots and the airplane. It is closely related to the distance at which the MSADM can respond. With the airplane cruising in the sky, it is always easy to find a direct and unobstructed path from the GCS to the airplane. Additionally, both the airplane and the GCS are able to be equipped with a long-range wireless communication module. Hence, the communication range of the MSADM is greatly increased compared to direct communication from the GCS to the ground robots.

The local communication network refers to the communication link between the ground robots and the airplane. Each agent, including the ground robots and the airplane, in the multi-robot troop is assigned with a unique ID and equipped with a commercial zigbee wireless module to build up the local communication network. Information gathered by each ground robot is able to be transmitted to the “airplane” node through the network as long as there exists a multi-hop path to the aerial robot, which greatly enhances the communication stability of the local network.

**Figure 6:**
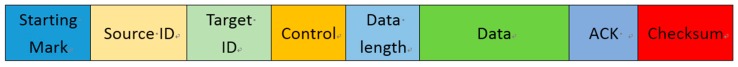
Format of frame.

To combine the local communication network and remote communication together, the message and data exchanged between GCS and ground robots is packed into frames following the protocol presented in Figure 6. Each node in the whole communication network (ground robots, airplane, GCS) is given a unique ID. The “Source ID” and “Target ID” indicate the sender and receiver of each frame. The “Checksum” byte is calculated by adding all the previous bytes in the frame to identify whether the frame has been successfully transported. The “ACK” is used here to identify whether an acknowledgement frame should be sent in response. The acknowledgement frame shares the same format as in Figure 6, except with a different two-byte “Starting mark”. The protocol treats the absence of a desired acknowledgement frame as communication lost. When the communication link is temporarily lost, the retransmission mechanism in the communication protocol enables the transmitter to resend the message if no corresponding answer is received 10 times. In such a situation, the gathered information is guaranteed to be buffered and transmitted to the ground control station.

With the two parts mentioned above, the proposed sensing mode is able to provide a longer communication range and more reliable communication performance than the traditional sensing mode in which the ground robots directly communication with GCS.

### 3.3. Modularity of the MSADM

**Figure 7:**
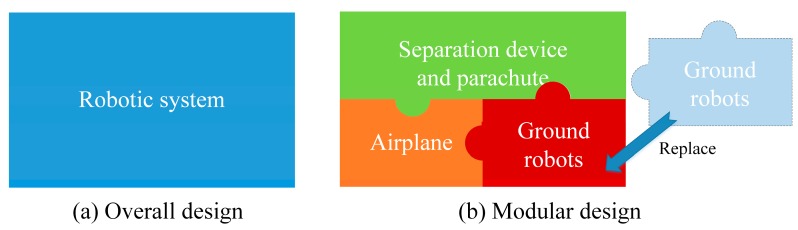
Overall design and modular design of the MSADM.

Modularity is another advantage of the MSADM. As is well known, a disaster robot is only able to collect some specific disaster information, depending on the sensors it is equipped with. This means that a robotic system must be modified when it is required to gather different information. However, traditional robotics systems which use the overall design principle need to be completely redesigned, because they integrate all the functions and sensors in a single robot. A possible way to avoid such problems is to use a modular design and divide the whole system into several modules with different functions. In this way, only parts of the modules need to be redesigned or replaced when the system is required to gather more disaster information (see Figure 7).

**Figure 8:**
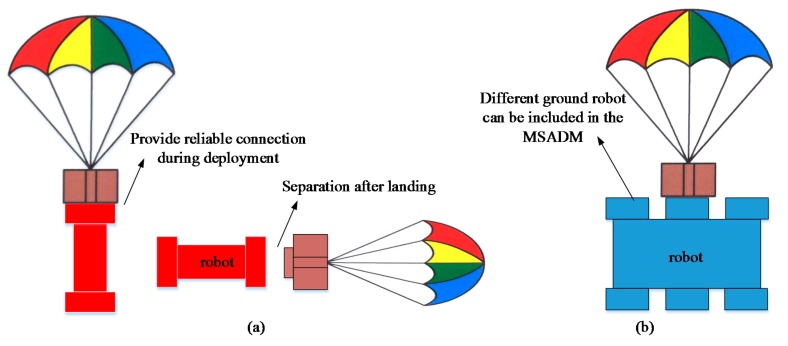
(**a**) Function of the separation device. (**b**) Any ground robot sharing the corresponding mechanical interface with separation device can quickly be included in the MSADM.

The MSADM uses a modular design approach and divides the system into several modules with different functions (See Figure 7). Briefly speaking, the airplane is used to overcome the paralyzed ground traffic problem, and ground robots are the searchers who ultimately collect the information. For the purpose of reducing the shock of the robot hitting the ground, a parachute is introduced to slow the robot’s velocity during deployment. The separation device connects the robot with the parachute during deployment, and separates itself from the ground robot (see Figure 8a). In particular, the separation device is designed to have a specific mechanical interface in order to connect with the ground robots. The advantage of this is that any ground robot which shares the corresponding mechanical interface with the separation device can quickly be included in the MSADM (see Figure 8b). In other words, ground robots with different functions can easily be replaced or integrated into the MSADM to gather different disaster information or perform different tasks.

## 4. Experimental Platform for Verification of the Proposed Sensing Mode

To verify the proposed disaster sensing mode, an experimental platform was developed by the authors. This section describes the components of the experimental platform used in the integrated experiment.

### 4.1. Airplane

**Figure 9:**
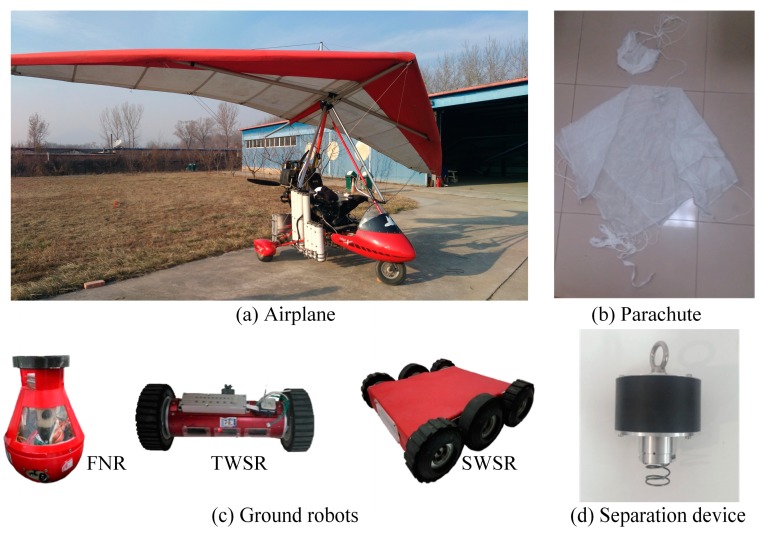
Experimental platform of MSADM.

In this system, an Airborne X-Series Classic Delta-wing aircraft is used as the airplane in MSADM (See Figure 9a). Powered by the Rotax 582DCDI engine, the airplane is able to cruise at a speed of 92 km/h with a maximum payload of up to 193 kg. To enable the transportation and deployment of ground robots, the airplane is equipped with a series of launch cabins. The launch cabin loads the ground robots during the transportation and opens the cabin door to deploy ground robots by rotating the motor. Using the communication link in Section 3.2, a long-range wireless serial communication module “MDS TransNET 900” and a zigbee wireless module WTL2422Z by “Cells-net” (Guangzhou, China) are responsible for the remote communication link and local communication network, respectively. In addition, a control board is responsible for relaying information between these two communication links and controlling the state of the cabin door. Therefore, the airplane can not only transport and deploy ground robots, but also relay information between the ground robots and the GCS.

### 4.2. Ground Robots

In an attempt to show the advantage of the modularity of MSADM, three different kinds of self-developed ground robots are used as ground robots in the experimental platform (see Figure 9c). These are the Fixed Node Robot (FNR), Two-Wheel Scout Robot (TWSR) and Six-Wheel Scout Robot (SWSR), and they are controlled in response to the commands received from the GCS. The FNR is a tumbler-like robot that cannot move around, but stands upright in a fixed position. TWSR and SWSR are mobile robots with different degrees of mobility and sensors. The specifications of these three robots are listed in Table 1. It is worth noting that the same mechanical interface is equipped on these three robots to connect to the separation device. In this way, various kinds of ground robots can be quickly replaced with each other and integrated into the MSADM, as long as they are equipped with this mechanical interface. 

### 4.3. Separation Device and Parachute

It is common knowledge that a free-falling object from the air is easily broken into pieces if it does not have special protection. Inspired by the paratrooper, the robots are equipped with a parachute to slow down the acceleration during falling and to prevent the robot from suffering a great shock which may damage the robot. Compatible with the three kinds of scout robot, two different-sized parachutes are custom manufactured in consideration of the aerodynamics and robot weights.

A self-developed separation device is used to connect the ground robot with the parachute during deployment, which separates itself from the ground robot after landing. The separation device has a control board integrated with a 3-axis accelerometer, a driving motor, and a modified slider-crank mechanism, and can identify the landing phase based on the measured acceleration.

To provide reliable separation control, an energy-based method is applied in the separation device to determine whether the robot has landed on the ground or not. In principle, the maximum energy loss occurs when the robot hits the ground. Using the impulse-momentum theorem in Equation (1), the energy of the object and the energy loss of an object can be expressed as Equations (2)–(4). In this way, the measured acceleration data can be transformed into the energy of the robot to find out the landing moment of the robot where the energy has the greatest change.

(1) mΔv=m∫adt=∫Fdt

(2) $$\Delta E_{i}=\frac{1}{2}m\Delta v^{2}=\frac{{\left(\int Fdt\right)}^{2}}{2m}=\frac{m{\left(\int adt\right)}^{2}}{2}$$

(3) $$E_{i}=\sum_{j=1}^{i-1}\Delta E_{j}$$

(4) $$E_{loss}(t)=E_{t-\Delta t}-E_{t}$$

**Figure 10:**
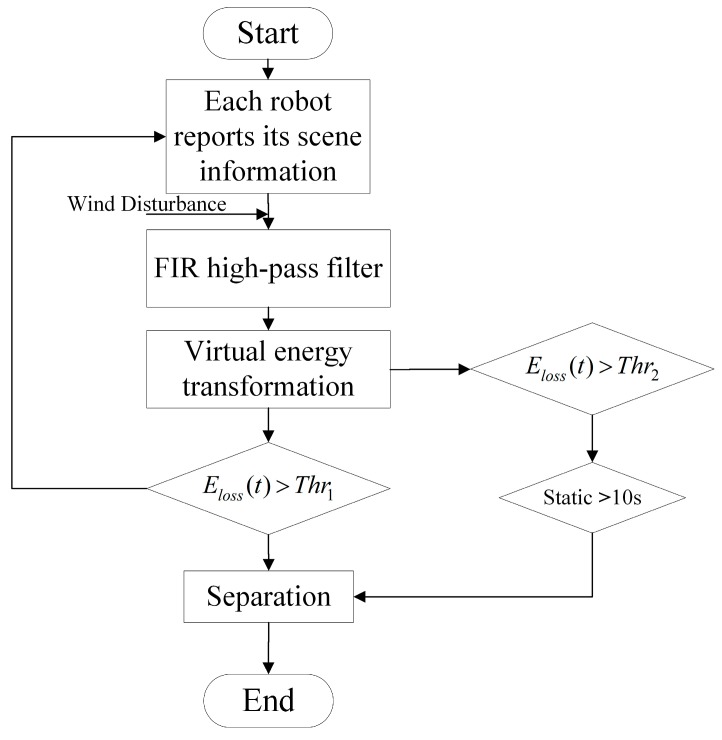
Flowchart of the separation control algorithm.

When considering an irregular wind, the robot may swing and accelerate in the air. The acceleration produced by the effect of the wind also contributes to the energy calculated in Equation (3) and may result in misidentification of the landing moment. Thus, the direct use of acceleration tends not to be robust. Fortunately, the unit impulse function shares a full frequency spectrum, while the constant acceleration and robot’s wobble caused by the wind is located on the lower part of the spectrum. Therefore, accelerometer data is passed through a high-pass filter before calculating the energy. In the system introduced in this paper, a fourth-order filter finite impulse response high-pass filter is used to avoid the influence of disturbance mentioned above, making our algorithm more robust. Another static mechanism is also utilized here by judging whether the robot has been static for 10 s. In practice, the cut-off frequency and two thresholds, ${Thr}_{1},{Thr}_{2}$, are selected by analyzing the experimental data. Figure 10 shows the flowchart of the separation control algorithm. With the above algorithm programmed into the separation controller, experiments were performed, verifying that the method can successfully separate the robot from the release device.

**Figure 11:**
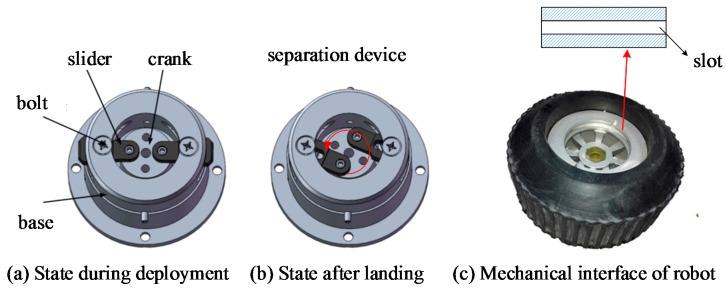
(**a**) States of the separation device during deployment. (**b**) States of the separation device after landing. (**c**) Corresponding mechanical interface of robots.

The slider-crank mechanism here is used to determine the connection state between the ground robot and the separation device. When the slider thrusts itself into the slot on the ground robot’s mechanical interface, the separation device and parachute are connected to the ground robot to slow down the robot’s speed during deployment (see Figure 11a). Once the device recognizes that the robot has landed on the ground, it rotates the crank, and the slider pulls out of the slot on the robot’s mechanical interface to separate ground robots from separation device (see Figure 11b). The robot must be attached to the separation device through the mechanical interface. For FNR, the mechanical interface is located on the top of the robot. For TWSR and SWSR, the mechanical interface of the robot is embedded in the wheel of the robot (see Figure 11c). An intuitive example of the connection and separation between the separation device with TWSR can be seen in Figure 8a.

## 5. Integrated Demonstration

In this section, the proposed sensing mode is implemented on the experimental platform and an integrated demonstration is presented to verify the effectiveness of the proposed sensing mode. The setup of the demonstration is introduced first, and the results are discussed at the end.

### 5.1. Demonstration Setup

For the purpose of implementing the proposed sensing mode, a disaster scenario similar to the Great Sichuan Earthquake, in which disaster blocked the ground traffic from the rescue center to the disaster site, is assumed by the authors. Based on the above scenario, an integrated demonstration was carried out in the independent airspace of Beijing Flying Base, Beijing, China using the experimental platform. In details, the airplane is required to carry 13 FNRs, 5 TWSRs, and 2 SWSRs and deploy them to the disaster site to collect ground image information.

**Figure 12:**
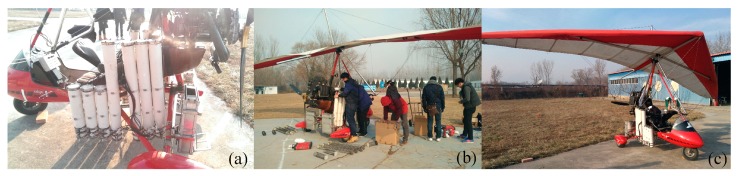
Preparation for the integrated demonstration: (**a**) Equip airplane with launch cabins. (**b**) Load ground robots, separation devices, and parachutes into launch cabins. (**c**) Preparation is done.

**Figure 13:**
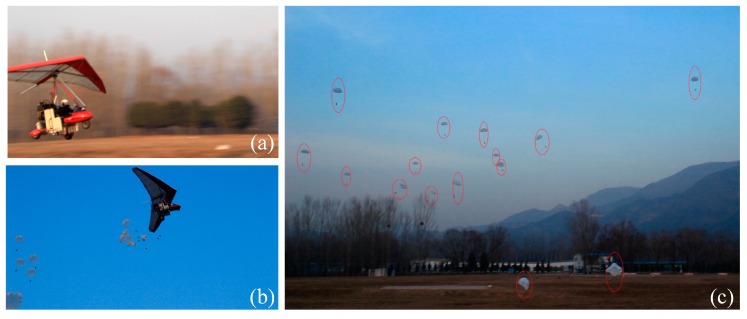
Deployment process of the integrated experiments: (**a**) The airplane took off from the ground. (**b**) The airplane deployed robots from the air. (**c**) Experimental scene of deployment.

Figure 12 shows the preparation for the integrated demonstration. The ground robots were connected to parachutes, along with separation devices, and loaded into the modified delta-wing aircraft. With all the preparation done, the experimental platform was fully ready for the disaster sensing task. After that, the seasoned pilot drove the airplane along the flight plan to simulate the long trip from the rescue center to the disaster site, and deployed the ground robots once the airplane had arrived at the destination (see Figure 13). Finally, the ground robots successfully landed and separated themselves from the separation device to collect disaster information.

### 5.2. Results

**Figure 14:**
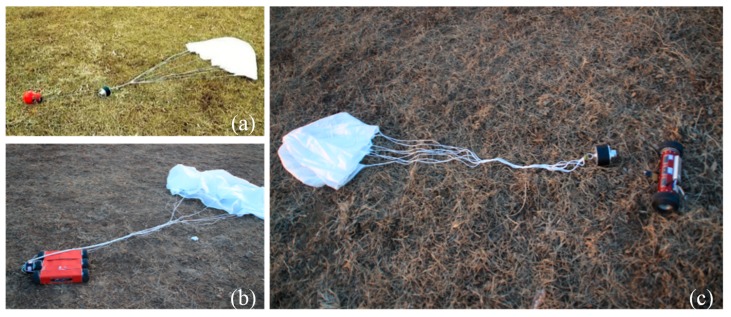
Successful landing and separation of robots: (**a**) FNR. (**b**) SWSR. (**c**) TWSR.

**Figure 15:**
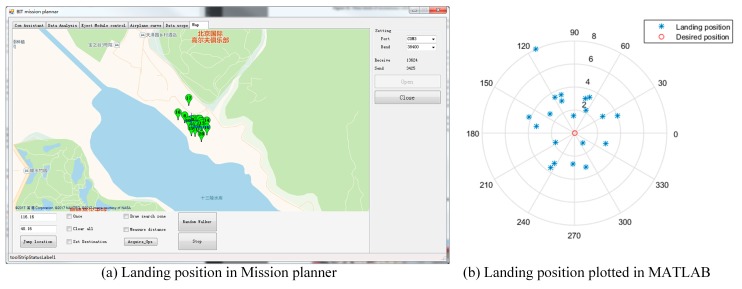
Landing position of the deployed ground robots.

All 20 robots landed successfully on the ground and separated themselves from their parachutes and separation device (see Figure 14). Image data and global position coordinates were used as the disaster environmental information in this experiment. They were gathered by the ground robots under the control of the GCS. Figure 15 shows the coordinates of the deployed ground robots reported to the GCS. The landing positions were scattered close to the desired destination under the influence of the wind during deployment. This shows that the ground robots can successfully be deployed to the disaster site using the proposed sensing mode. It is worth noting that the robotic platform accesses the disaster site from the air without considering the ground traffic, which indicates that the proposed sensing mode is still effective even when ground traffic from the rescue center to the disaster site is disrupted.

**Figure 16:**
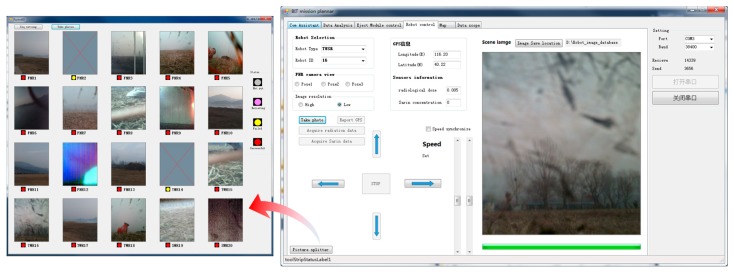
Disaster image information collected by the ground robots.

Figure 16 shows the image data collected by the ground robots. The GCS was set at a location 3 km away from the assumed disaster site, and there was no direct line-of-sight communication path between the destination and the GCS location. The communication performance benefits from the relay mechanism in Section 3.2, which transforms the blocked ground communication link between the GCS and ground robots into two barrier-free ground-air communication components. Thus, the effectiveness of the communication performance was strengthened.

It is notable is that three kinds of robot were successfully deployed to the disaster site and collected disaster information in this demonstration. Apart from having the same mechanical interface to connect to the separation device, they were of different sizes and had different properties. These results support the view of the modular design concept in Section 3.3. The advantage of this property is that various robots, not limited to the three above, can be quickly integrated into the MSADM simply by modifying the mechanical interface. Therefore, the proposed sensing mode can use robots with different sensors and functions tailored towards different disaster events.

### 5.3. Comparison with Other Sensing Modes

In this section, the proposed sensing mode using MSADM is compared with the traditional sensing mode, which uses ground or aerial robots only (see Table 2). The traditional sensing mode using ground robots only can be analyzed by assuming there is no airplane in Section 5.1. The most intuitive result of using this sensing mode is that the ground robots are unable to reach the destination due to the paralyzed ground traffic. In addition, the direct communication between the ground robots and the GCS easily suffers due to the obstacles between them.

The traditional sensing mode using aerial robots independently can be easily simulated in the integrated demonstration by imagining there are no ground robots in the MSADM. The airplane has the advantages of fast speed and an unimpeded path through the air, allowing it to quickly reach the disaster site in paralyzed ground traffic situations. However, the aircraft here can only provide a bird’s eye view of disaster scenes from the air, rather than collecting detailed image views on the ground like a ground robot. Furthermore, there is also some information that is more suitable to be observed from the ground, such as the concentration of chemical pollution in the soil after a chemical spill. Small aerial robots can fly at low altitude to give a clearer view of the ground, but they are unsuitable for covering long distances, and operating over a long time, due to their short endurance time and their requirement of more precise control to avoid damage.

In the proposed mode using MSADM for disaster sensing, the advantages of the unimpeded air traffic of the aerial robots are combined with the direct detailed view. In addition, with the airplane cruising in the sky and acting as a relay, the communication performance was established. Finally, ground robots with different functions can be easily replaced or integrated into the MSADM to gather different disaster information or to perform different tasks.

## 6. Summary and Conclusions

This paper presents a disaster sensing mode using MSADM to collect disaster ground information when the ground traffic from the rescue center to disaster site is disrupted. The main idea of the MSADM is to combine the unimpeded air traffic of the aerial robots with the direct detailed view of the ground robots in an integrated robotic system. In detail, the MSADM uses an airplane to carry some miniature ground robots to overcome the paralyzed traffic problem and deploys them on the ground to collect detailed scene information. The experiment results demonstrated the effectiveness of the proposed sensing mode.

There are several advantages, as compared to the traditional sensing mode using aerial or ground robots independently. Firstly, the proposed sensing mode is more suitable for collecting detailed disaster ground information when the ground traffic from the rescue center to disaster site is disrupted. In detail, the MSADM is able to access the disaster destination more quickly through the air, compared to ground robots, and is able to collect more detailed information on the ground than aerial robots. Secondly, the communication performance is established by using the airplane in the sky to relay information between the ground robots and the GCS. By doing this, the blocked ground communication link between the GCS and ground robots has been transformed into two barrier-free ground–air communication components. Last, but not least, the modular design enables different ground robots to be quickly replaced or integrated into the MSADM, as long as they share a specific mechanical interface, which means that the proposed mode can be applied to gathering different disaster information in different events.

In the future work, more attention will be paid to the coordination control of the deployed ground robots to improve the disaster sensing task. Furthermore, MSADM consisting of more kinds of robots, such as small drones, legged robots, and robots with manipulation ability, should be considered, to provide a more robust performance towards different disaster situations.

## Figures and Tables

**Table 1:** Specification of the ground robots.

Item	FNR	TMSR	SWSR
Mass	0.48 kg	1.6 kg	7.33 kg
Size	9.5 × 9.5 × 13.7 cm	10 × 10 × 28 cm	34.2 × 36.7 × 9.6 cm
Maximum Speed	0	0.6 km/h	1.2 km/h
Sensors	Camera, GPS	Camera, GPS, IMU, etc.	Camera, GPS, IMU, etc.
Radio communication	Zigbee	Zigbee	Zigbee

**Table 2:** Comparison with other sensing modes.

	Using Aerial Robots	Using Ground Robots	Using the MSADM
Conquer the paralyzed ground traffic	Yes	No	Yes
Collected detailed ground information	Bird’s eye view from the air only.	Detailed	Detailed
Communication path	Ground to air.	Ground to ground. Easily blocked by the obstacles between GCS and robot.	Transform the blocked ground to ground path into two barrier-free ground-air communication paths.
Scalability	--	--	Different ground robots can be quickly integrated in the MSADM.

## References

[1] Suzuki T. (2008). Field test on the application of disaster mitigating information sharing platform to local governments and its evaluation. J. Disaster Inf. Stud..

[2] Siciliano B., Khatib O. (2016). Springer Handbook oƒ Robotics.

[3] Murphy R.R. (2004). Trial by fire [rescue robots]. IEEE Robot. Autom. Mag..

[4] Juang S.-Y., Juang J.-G. (2016). Remote control of a mobile robot for indoor patrol. Appl. Sci..

[5] Goodrich M.A., Morse B.S., Engh C., Cooper J.L., Adams J.A. (2009). Towards using Unmanned Aerial Vehicles (UAVs) in Wilderness Search and Rescue: Lessons from field trials. Interact. Stud..

[6] Goodrich M.A., Cooper J.L., Adams J.A., Humphrey C., Zeeman R., Buss B.G. Using a mini-UAV to support wilderness search and rescue: Practices for human-robot teaming. Proceedings of the SSRR2007—IEEE International Workshop on Safety, Security and Rescue Robotics.

[7] Goodrich M.A., Morse B.S., Gerhardt D., Cooper J.L., Quigley M., Adams J.A., Humphrey C. (2008). Supporting wilderness search and rescue using a camera-equipped mini UAV. J. Field Robot..

[8] Sun J., Li B., Jiang Y., Wen C.Y. (2016). A camera-based target detection and positioning UAV system for search and rescue (SAR) purposes. Sensors.

[9] Ambrosia V.G., Wegener S., Zajkowski T., Sullivan D.V., Buechel S., Enomoto F., Lobitz B., Johan S., Brass J., Hinkley E. (2011). The Ikhana unmanned airborne system (UAS) western states fire imaging missions: From concept to reality (2006–2010). Geocarto Int..

[10] Szynkarczyk P., Czupryniak R., Trojnacki M., Andrzejuk A. Current state and development tendency in mobile robots for special applications. Proceedings of the International Conference WEISIC 6th Workshop on European Scientific and Industrial Collaboration on Promoting Advanced Technologies in Manufacturing.

[11] Barnes M., Everett H.R., Rudakevych P. ThrowBot: Design considerations for a man-portable throwable robot. Proceedings of the Unmanned Ground Vehicle Technology VII, International Society for Optics and Photonics.

[12] Lester J., Brown A., Ingham J.M. Christchurch cathedral of the blessed sacrament: Lessons learnt on the stabilisation of a significant Heritage Building. Proceedings of the 2012 New Zealand Society of Earthquake Engineering.

[13] Kruijff G.-J.M., Pirri F., Gianni M., Papadakis P., Pizzoli M., Sinha A., Tretyakov V., Linder T., Pianese E., Corrao S. Rescue robots at earthquake-hit Mirandola, Italy: A field report. Proceedings of the 2012 IEEE International Symposium On Safety, Security, And Rescue Robotics (SSRR).

[14] Wang W., Dong W., Su Y., Wu D., Du Z. (2014). Development of search-and-rescue robots for underground coal mine applications. J. Field Robot..

[15] Zhao J., Gao J., Zhao F., Liu Y. (2017). A search-and-rescue robot system for remotely sensing the underground coal mine environment. Sensors.

[16] Voyles R.M., Larson A.C. (2005). TerminatorBot: A novel robot with dual-use mechanism for locomotion and manipulation. IEEE/ASME Trans. Mechatron..

[17] Campbell D., Buehler M. (2003). Stair descent in the simple hexapod “RHex”. IEEE Int. Conf. Robot. Autom..

[18] Ezequiel C.A.F., Cua M., Libatique N.C., Tangonan G.L., Alampay R., Labuguen R.T., Favila C.M., Honrado J.L.E., Canos V., Devaney C. UAV aerial imaging applications for post-disaster assessment, environmental management and infrastructure development. Proceedings of the 2014 International Conference on Unmanned Aircraft Systems (ICUAS).

[19] Erdelj M., Natalizio E. UAV-assisted disaster management: Applications and open issues. Proceedings of the 2016 International Conference on Computing, Networking and Communications, ICNC.

[20] Hsieh M.A., Cowley A., Keller J.E., Chaimowicz L., Grocholsky B., Kumar V., Taylor C.J., Endo Y., Arkin R.C., Jung B. (2007). Adaptive teams of autonomous aerial and ground Robots for situational awareness. J. Field Robot..

[21] Michael N., Shen S., Mohta K., Kumar V., Nagatani K., Okada Y., Kiribayashi S., Otake K., Yoshida K., Ohno K. (2014). Collaborative mapping of an earthquake damaged building via ground and aerial robots. Springer Tracts in Advanced Robotics.

[22] China Parachutes 100 Soldiers to Cut-Off Quake Area. https://reliefweb.int/report/china/china-parachutes-100-soldiers-cut-quake-area.

[23] Li B., Jiang Y., Sun J., Cai L., Wen C.Y. (2016). Development and testing of a two-UAV communication relay system. Sensors.

